# Genetic variation, population structure and linkage disequilibrium in peach commercial varieties

**DOI:** 10.1186/1471-2156-11-69

**Published:** 2010-07-20

**Authors:** Maria José Aranzana, El-Kadri Abbassi, Werner Howad, Pere Arús

**Affiliations:** 1 IRTA. Centre de Recerca en Agrigenòmica CSIC-IRTA-UAB, Carretera de Cabrils Km 2, 08348 Cabrils (Barcelona), Spain

## Abstract

**Background:**

Peach [*Prunus persica *(L.) Batsch] is one of the most economically important fruit crops that, due to its genetic and biological characteristics (small genome size, taxonomic proximity to other important species and short juvenile period), has become a model plant in genomic studies of fruit trees. Our aim was an in-depth study of the extent, distribution and structure of peach genetic variation in North American and European commercial varieties as well as old Spanish varieties and several founders used in the early USA peach breeding programmes. For this we genotyped 224 peach cultivars using 50 SSRs evenly distributed along the 8 linkage groups of the *Prunus *reference map.

**Results:**

Genetic distance analysis based on SSRs divided the peach cultivars in three main groups based mainly on their fruit characteristics: melting flesh peaches, melting flesh nectarines and non-melting varieties. Whereas non-melting flesh peaches had a higher number of alleles than melting peaches and nectarines, they were more homozygous. With some exceptions ('Admiral Dewey', 'Early Crawford' and 'Chinese Cling'), the founder US cultivars clustered together with the commercial melting peaches, indicating that their germplasm is well represented in modern cultivars. Population structure analysis showed a similar subdivision of the sample into subpopulations. Linkage disequilibrium (LD) analysis in three unstructured, or barely structured, subpopulations revealed a high level of LD conservation in peach extending up to 13-15 cM.

**Conclusions:**

Using a much larger set of SSRs, our results confirm previous observations on peach variability and population structure and provide additional tools for breeding and breeders' rights enforcement. SSR data are also used for the estimation of marker mutation rates and allow pedigree inferences, particularly with founder genotypes of the currently grown cultivars, which are useful to understand the evolution of peach as a crop. Results on LD conservation can be explained by the self-pollinating nature of peach cultivated germplasm and by a bottleneck that occurred at the beginning of modern breeding practices. High LD suggests that the development of whole-genome scanning approaches is suitable for genetic studies of agronomically important traits in peach.

## Background

Peach is the most important of the stone fruit crops, that also include plum (*P. domestica *and *P. salicina*), apricot (*P. armeniaca*) and cherry (*P. avium *and *P. cerasus*). It originated in China where it was domesticated 4-5,000 years ago [1]. Its cultivation extended to central Asia and later to Europe where it is known to have been cultivated by the Romans. It was taken from Europe to the American continent with the first Spanish colonizers around 500 years ago. For centuries, peach was cultivated and selected for different agronomical characters, leading to locally adapted populations. After the rediscovery of Mendel's laws and their impact on the development of modern breeding programs, North American breeders started, about 75 years ago, to produce a new wave of varieties. These were based on a small number of founder cultivars, mainly accessions of European origin plus at least one Chinese accession ('Chinese Cling'). These breeding programs were extremely successful and most commercial varieties grown today in America and Europe descend from them.

Microsatellite or simple-sequence repeat (SSR) markers, have been very useful for studying the extent and distribution of genetic variability in wild and cultivated plants including various *Prunus *species [2-4]. Our results from genotyping 212 peach cultivars with a set of 16 unmapped SSRs [5] indicated that these markers can be used to individually identify most genotypes and classify the cultivars according to key morphological attributes (mainly peaches, nectarines and non-melting flesh peaches). We also found that certain breeding history elements from old seed-propagated varieties were crucial in the observed variability, as cultivars from modern breeding programmes are more heterozygous.

In this paper we re-examine this collection of genotypes, with the addition of several American founder accessions, with a set of 50 SSRs that cover the peach genome [6]. With these markers we analyze in more depth the population parameters of the previous work [5] and study other aspects of peach variability and genome organization including subpopulation structure. This research also provides a first insight into linkage disequilibrium conservation in peach. Unlike other *Prunus *species (almond and other stone fruit) that have an effective gametophytic self-incompatibility system, peach is self-fertile. The outbreeding rate of peach has been estimated to be ~15% [7], implying that there is a 4-fold reduction in its effective recombination rate, compared to an obligate outcrossing species [8]. Self-fertilization, a major germplasm bottleneck due to its recent breeding history [1,9], and the fact that peach is a long lived species that can be vegetatively reproduced allowing for long intergenerational periods, indicate that conservation of linkage disequilibrium (LD) may be as high as in species that share one or more of these features [10-12]. Understanding the patterns of LD across the genome in the available germplasm, as one of the key genetic features of peach, will help choose the appropriate methodology for genetic association mapping.

## Results

### Genetic Variability Analysis

The 50 SSRs studied amplified 318 alleles, an average of 6.36 alleles per locus (A) and 2.08 the average effective number of alleles (Ae) (Table 1). As indicated by the difference between A and Ae, a large proportion of these alleles (52.2%) had frequencies lower than 5%, with 31.6% of them (43 alleles) present in a single genotype. Only two of the 8 founders had alleles not present in the group of commercial varieties, 'Chinese Cling' (3 alleles) and its seedling 'Elberta' (1 allele, inherited from the 'Chinese Cling' parent). The founder varieties contained 43% of the alleles present in the contemporary ones.

**Table 1:** Variability parameters calculated for 50 SSR markers in 224 peach cultivars

SSR	A	Ae	Ho	He	F	# Genotypes	PD	Reference
BPPCT001	9	3.46	0.55	0.71	0.22	26	0.87	[37]
BPPCT006	11	2.77	0.50	0.64	0.22	21	0.81	[37]
BPPCT007	7	2.40	0.46	0.58	0.21	13	0.75	[37]
BPPCT008	11	1.55	0.18	0.36	0.50	24	0.45	[37]
BPPCT014	5	1.55	0.32	0.36	0.10	7	0.52	[37]
BPPCT015	15	3.11	0.57	0.68	0.16	27	0.85	[37]
BPPCT017	9	2.20	0.42	0.55	0.24	13	0.73	[37]
BPPCT020	6	2.69	0.39	0.63	0.38	12	0.78	[37]
BPPCT024	5	1.16	0.14	0.14	-0.01	6	0.25	[37]
BPPCT025	10	3.09	0.44	0.68	0.35	24	0.82	[37]
BPPCT037	7	2.11	0.43	0.53	0.18	11	0.68	[37]
BPPCT038	9	2.12	0.41	0.53	0.23	16	0.67	[37]
BPPCT039	2	1.65	0.37	0.39	0.06	3	0.56	[37]
CPPCT002	3	2.04	0.31	0.51	0.39	6	0.66	[38]
CPPCT005	8	2.50	0.45	0.60	0.26	15	0.78	[38]
CPPCT006	3	2.19	0.42	0.54	0.24	6	0.72	[38]
CPPCT013	3	1.02	0.02	0.02	-0.01	3	0.03	[38]
CPPCT015	4	1.07	0.06	0.06	0.12	5	0.11	[38]
CPPCT022	10	5.04	0.56	0.80	0.30	26	0.92	[38]
CPPCT026	11	1.91	0.26	0.48	0.46	18	0.62	[38]
CPPCT027	4	1.36	0.11	0.26	0.60	8	0.35	[38]
CPPCT029	7	1.98	0.44	0.50	0.10	12	0.68	[38]
CPPCT030	8	2.72	0.52	0.63	0.17	17	0.81	[38]
CPPCT033	5	2.16	0.32	0.54	0.40	10	0.71	[38]
CPPCT040	6	1.89	0.27	0.47	0.43	13	0.63	[6]
CPPCT042	6	2.06	0.43	0.52	0.17	13	0.71	[6]
CPPCT044	10	3.40	0.55	0.71	0.22	22	0.87	[6]
CPPCT046	4	1.51	0.33	0.34	0.01	5	0.53	[6]
CPSCT006	5	1.14	0.09	0.12	0.24	8	0.20	[39]
EPPCU1090	5	1.87	0.40	0.47	0.13	9	0.66	[21]
PCeGA34	4	1.74	0.26	0.42	0.38	6	0.58	[40]
pchcms5	3	1.68	0.33	0.41	0.20	6	0.59	[41]
pchgms1	3	1.02	0.01	0.02	0.50	4	0.03	[41]
pchgms2	3	1.18	0.14	0.15	0.03	4	0.26	[41]
pchgms3	6	1.33	0.20	0.25	0.19	10	0.40	[41]
pchgms6	8	3.40	0.52	0.71	0.26	15	0.86	[41]
PMS2	6	1.15	0.09	0.13	0.31	10	0.20	[42]
PS1H3	3	1.86	0.38	0.46	0.17	4	0.63	[42]
PS9f8	8	2.04	0.43	0.51	0.15	12	0.71	[43]
UDP96-001	6	1.49	0.19	0.33	0.44	11	0.45	[44]
UDP96-003	10	2.73	0.52	0.63	0.17	19	0.80	[44]
UDP96-005	7	1.97	0.36	0.49	0.28	15	0.65	[44]
UDP96-008	5	1.91	0.35	0.48	0.26	9	0.64	[44]
UDP96-013	7	1.67	0.22	0.40	0.44	12	0.55	[44]
UDP96-015	8	2.81	0.42	0.64	0.34	24	0.79	[44]
UDP96-018	5	2.03	0.36	0.51	0.28	9	0.69	[44]
UDP97-401	3	1.56	0.21	0.36	0.41	4	0.51	[44]
UDP98-024	5	2.81	0.47	0.64	0.28	11	0.82	[45]
UDP98-025	5	2.44	0.42	0.59	0.28	11	0.77	[45]
UDP98-409	5	1.64	0.26	0.39	0.33	9	0.56	[44]

**Average**	6.36	2.08	0.34	0.46	0.26	12.08	0.60	

A = number of observed alleles; Ae = effective number of alleles; Ho = observed heterozygosity; He = expected heterozygosity; F = Wright's fixation index; PD = power of discrimination.

We observed an average SSR heterozygosity (Ho) of 0.34, while the expected heterozygosity (He) was 0.46. Consequently, the fixation index (*F*) values were positive, with a mean of 0.26 for all loci. The average of power of discrimination (PD) was 0.60, with CPPCT022 being the marker with the highest ability to discriminate between two random cultivars (PD = 0.92). The probability of confusion calculated using the PD values, was extremely low: C = 3.32 × 10^-24^.

**Figure 1:**
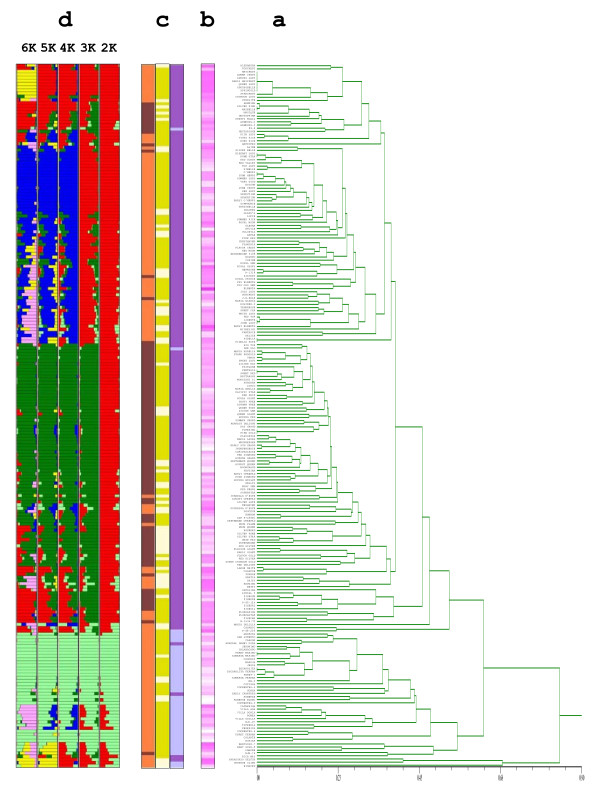
**Diversity analysis and population structure of 224 peach cultivars using 50 SSRs loci**. a) Dendrogram based on the genetic distance; b) observed heterozygosity, Ho, of each cultivar; color intensity increases with increase in Ho; c) Fruit characteristics: orange = peach, brown = nectarine, pistachio = yellow flesh, white = white flesh, purple = melting, pale lilac = non-melting; d) population stratification for K = 2 to K = 6 (each color represents a different subpopulation)

**Figure 2:**
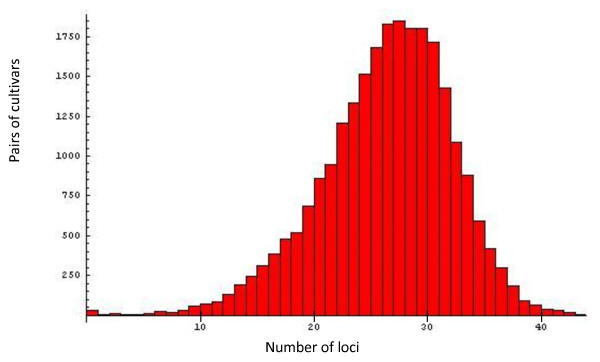
**Distribution of the number of SSR loci differing between pairs of peach cultivars**.

The number of different genotypes amplified by each marker ranged from 3 (BPPCT039 and CPPCT013) to 27 (BPPCT015) with an average of 12.1. The whole set of 50 SSRs used was able to identify 209 different genotypes among the 224 cultivars studied (Figure 1a). Ten groups of cultivars (1 group with 7 cultivars and 9 with 2 cultivars each) had identical genotypes. These sets had been previously detected [5] with 16 SSRs. The number of locus differences between each possible pair of cultivars ranged from 0 to 43 (i.e. two randomly chosen varieties may differ in up to 43 of the 50 studied loci), with an average of 25 (50% of the SSRs studied). The distribution of genotype differences between pairs of cultivars (Figure 2) shows that only a very small percentage of pairs of cultivars (0.3%), including all groups of known sports (cultivars originated by somatic mutation), differ at 6 or less loci.

The percentage of heterozygous loci at each cultivar ranged from 0 to 79.4%, with an average of 33.4%. Three cultivars had all the SSR loci tested in homozygosis: the non-melting cultivars 'Cofrentes-6' and 'Auberge Blanc', and the nectarine 'Independence', whereas 'Elberta' had the highest rate of heterozygosis. These values are shown in Additional file 1 and in Figure 1b, where these results were plotted against the distance tree, indicating Ho in pink color (color intensity increases with Ho values).

A tree constructed from the SSR data divided the cultivars into clusters characterized by correspondence with fruit characteristics: melting peach/nectarine/non-melting peach (Figure 1c). These morphological characteristics, i.e. melting/non-melting and nectarine/peach are each determined by a single gene [13]. The dendrogram placed most of the melting varieties (97.3%) in a single cluster, distinguishing 2 sub-clusters, one mainly for peaches and one for most nectarines, whereas most non-melting varieties clustered in an undefined and more diverse group. Five of the founders ('Elberta', 'Fay Elberta', 'Early Elberta', 'Rio Oso Gem' and 'J.H. Hale') grouped together with the modern melting peaches. The 'Admiral Dewey', 'Early Crawford' and 'Chinese Cling' founder varieties were positioned in the more diverse cluster together with the non-melting cultivars.

### Genetic variability in cultivar subsets

We calculated the variability in the 3 main groups, separated by fruit morphology: melting peaches, melting nectarines and non-melting peaches. Given that these groups have different sample sizes, we compared the number of alleles per cultivar (Ai) as shown in Table 2. The non-melting peaches amplified a much larger number of alleles per cultivar (A_i _= 5.54) than melting peaches (A_i _= 2.65) and nectarines (A_i _= 2.46). In contrast these varieties were more homozygous (Ho = 0.26 compared to 0.39 in melting peaches and 0.31 in nectarines). The proportion of unique alleles was similar in melting peaches and nectarines (10.8% and 11.6% respectively) and higher in non-melting peaches (15.7%).

**Table 2:** Variability for 50 SSRs in different peach cultivar subsets

	# Cultivars	A	**A**_**i**_	Ae	**Ae**_**i**_	Ho	He	F	# Genotypes
**All cultivars**	224	318	1.42	104.18	0.47	-	-	-	209
**Melting peaches**	94	249	2.65	99.42	1.06	0.39	0.43	0.11	85
**Nectarines**	91	224	2.46	88.95	0.98	0.31	0.36	0.15	88
**Non-melting peaches**	39	216	5.54	107.47	2.76	0.26	0.42	0.31	37

A = number of observed alleles; A_i _= number of alleles/number of cultivars; Ae = effective number of alleles; Ae_i _= effective number of alleles/number of varieties; Ho = observed heterozygosity; He = expected heterozygosity; F = Wright's fixation index.

### Population Structure

The cultivar collection was evaluated for population stratification. We analyzed the data by successively increasing the number of subpopulations (*K*) from two to eight. With *K *= 2 we detected a subdivision between melting and non-melting cultivars. Moving to *K *= 3, the cluster of melting cultivars split into two subpopulations, one principally peaches and the other, nectarines. With increasing *K*, three populations remained almost invariable (in blue, dark green and light green with *K *= 6, Figure 1d), whereas that in red divided into smaller subpopulations. Sorting these results in parallel with the dendrogram, there is clear agreement between population subdivision and genetic diversity (Figure 1d).

Based on these results we can consider three unstructured populations: MP with 25 melting peaches (blue), N with 50 nectarines (dark green) and NMP with 21 non-melting peaches (light green). An accession was considered to belong to a population if its membership coefficient was ≥ 80% in *K *= 6.

### Linkage Disequilibrium

It is known that population structure increases linkage disequilibrium (LD) in the genome. For this reason we calculated the LD for each of the three unstructured subpopulations described. After removing low frequency alleles (considering MAF ≥ 0.05), we calculated interallelic r^2^, i.e. the association between each of the alleles at the first locus and at the second one. A total number of 4,306, 5,814 and 3,301 pairs of alleles were compared in the MP, N and NMP subpopulations, respectively, with 515 in MP, 649 in N and 444 in NMP being placed in the same linkage group (intra-chromosome comparisons).

**Figure 3:**
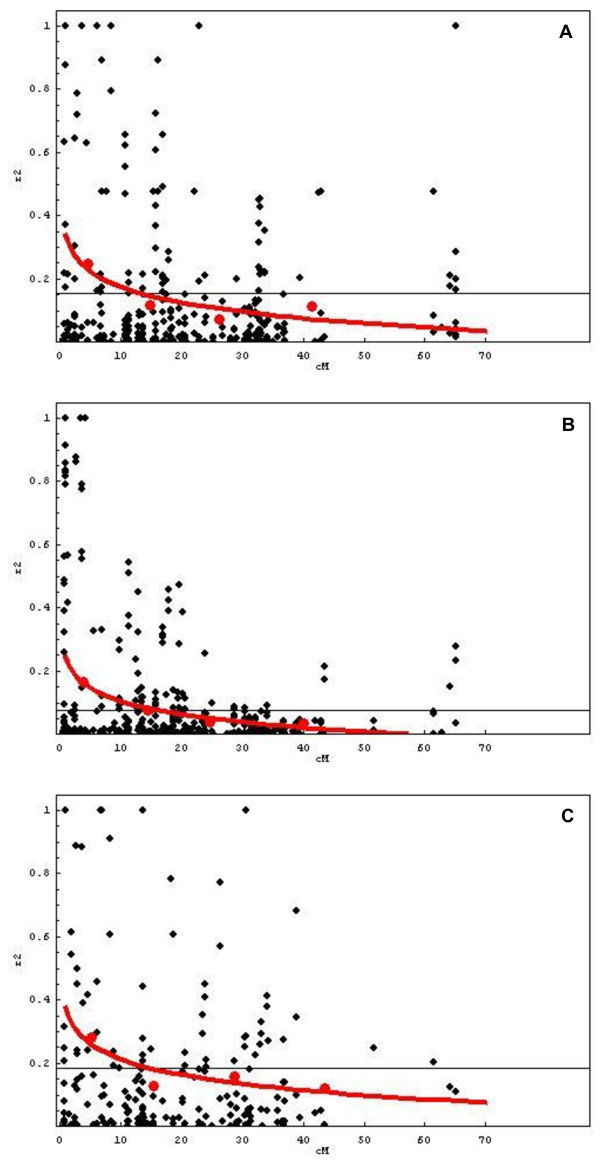
**Variation of LD against genetic distance**. The variation with distance (cM) of r^2 ^values between alleles of markers placed in the same linkage group in 25 melting peaches (A), 50 nectarines (B) and 21 non-melting peaches (C). Horizontal line indicates significance threshold at α ≤ 0.05. The average r^2 ^values at each of 4 subsets of equal number of pair comparisons covering adjacent intervals (red dots) were used to calculate the curve (red line) that represents how observed r^2 ^decreases with distance between pairs of markers.

In Figure 3 we show the variation of r^2 ^values plotted against distance. In all three populations, the LD clearly decays with distance. LD in melting peaches rapidly declines as pairs of SSRs become more distant, whereas in both nectarines and non-melting peaches, this is slower. The curves that describe the variation of r^2 ^with distance show that it reaches significant values at 13.3 cM in MP (r^2 ^≥ 0.15), 15.9 cM in N (r^2 ^≥ 0.07), and 15.2 cM in NMP (r^2 ^≥ 0.18). These distances decrease considerably at a significance threshold of α ≤ 0.01 (2.8 cM in MP, 6.3 cM in N and 2.3 cM in NMP).

**Figure 4:**
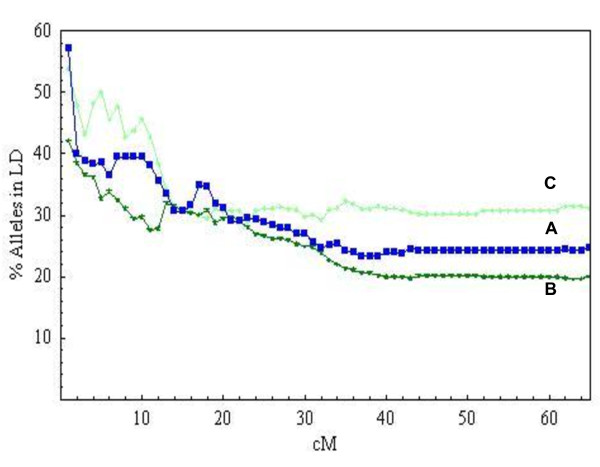
**Variation in percentage of pairs of alleles in LD with genetic distance**. The points indicate the proportion of pairs of alleles of markers placed in the same linkage group in significant LD (α ≤ 0.05) in 25 melting peaches (A), 50 nectarines (B) and 21 non-melting peaches (C).

The percentage of allele comparisons with significant LD between pairs of alleles of SSRs mapped in different linkage groups (unlinked comparisons) was 13.9% in the melting peach population, 13.4% in nectarines and 18% in non-melting peaches. When considering intra-chromosome comparisons, these figures rose to 25%, 20% and 31%, respectively, with the ratios decreasing with distance in all three cases. The variation with genetic distance of the percentage of pairs of alleles in LD for markers mapped in the same linkage group in each of the 3 subpopulations is shown in Figure 4. We observed that, in the peach population, the proportion of pairs of alleles in LD plummeted at very short distances, from 57% at 1 cM to 40% at 2 cM, stabilised up to 11 cM, then decreased dramatically again up to 14 cM to finally reach a plateau at about 35 cM. In nectarines, there was a steady decrease in pairs of alleles in LD, from 42% at 1 cM to 28% at 11 cM, followed by a slower decline, to reach a plateau around 30 cM. There was an overall but fluctuating decrease for non-melting peaches, reaching a plateau at a distance of 14 cM.

In all three populations we observed a region, from 1 to 11-14 cM, where the LD decays considerably with distance, then slowly reaching a constant level.

## Discussion

### SSR variability

We genotyped 224 peach and nectarine cultivars with 50 SSR markers covering the whole *Prunus *genome. Most of the varieties (208) used here coincide with the 212 previously genotyped in [5] with fewer markers (16 SSRs). There we found a larger number of alleles per locus (7.3 vs. 6.36) and a similar Ho (0.35 vs. 0.34). This reduction in allele number seems to be related to lower overall polymorphism of the new markers, given that the 16 new cultivars sampled do not appear to be less variable. On the other hand, the pairwise distance between the markers of the same linkage group ranged from 1 to 65 cM, with 40.4% of them being less than 14 cM apart. LD analysis showed that around 30-60% of the pairs of alleles of SSRs less than 11-14 cM apart may be in LD, and consequently much of the information from the markers used is not independent. A genotyping set with 24 SSR markers 20-25 cM apart covering the whole *Prunus *genome was previously proposed [14]. Here we have used 20 of these 24 proposed markers (all but BPPCT028, CPPCT016, CPPCT017 and UDP98-412), which yielded an average of 7.15 alleles per marker with an Ho of 0.37 in our sample of 224 cultivars, higher than with the overall set of 50 markers. The dendrogram constructed with this reduced set of markers was very similar to that constructed with the whole set of SSRs (not shown). This indicates that, for variability analysis or cultivar fingerprinting studies, the genotyping set is sufficient and that, in most cases, the use of additional markers would not produce an effective increase in relevant information.

### Genetic comparison between ancient and new cultivars

Most of the modern North American and European cultivars have originated from those produced in the US breeding programs of the early twentieth century, using a reduced number of parents as founders [1,9,15]. Eight of these were studied with the aim of exploring how much of their genome is represented in modern germplasm. We only observed a few unique alleles in 'Chinese Cling' and its seedling 'Elberta', whereas 43% of the alleles present in the modern cultivars were also present in the old varieties. The distribution of the founders in the dendrogram and their population subdivision suggests that five of them ('Elberta', 'Fay Elberta', 'Early Elberta', 'Rio Oso Gem' and 'J.H. Hale') are a major and direct source of current peach variability. The three remaining founders ('Admiral Dewey', 'Early Crawford' and 'Chinese Cling') cluster with the most diverse group (non-melting peaches), suggesting that they may have participated to a lesser extent in the creation of the new peach varieties. The fact that 'Admiral Dewey' and 'Early Crawford', with Ho values lower than average (0.11 and 0.29, respectively), cluster with a group of highly inbred Spanish peaches is consistent with their being closely related to old European varieties. 'Early Crawford' is considered the most important early peach in the last half of the 19th century and a standard of fruit quality [15], however genetic data show its divergence with modern cultivars. It is possible that it and 'Chinese Cling' (parent of 'Elberta') were used as initial parents, with other genotypes more broadly used in later breeding programs.

None of the founders grouped together with the nectarine varieties, supporting an earlier hypothesis [5] that different genetic resources were used in early breeding programs to obtain peaches and nectarines. This would explain the high molecular differentiation among varieties with these two fruit morphologies that are based on a single gene mutation.

### Genetic Variability in Cultivar Subsets

In agreement with previous results [5], the dendrogram and population structure analysis divided the sample into three major groups, one largely integrated by melting-peaches, one by nectarines and the third by non-melting peaches. When separating the 224 cultivars by these three fruit characteristics, we observed that melting peaches and nectarines had similar numbers of alleles, whereas the non-melting cultivars, most of them local Spanish varieties that have been traditionally self propagated [16], are more diverse but with higher levels of homozygosis. This may be due to the different strategies employed: breeding melting commercial cultivars is usually by crossing two individuals and selecting from their progeny, while non-melting peaches, particularly Spanish varieties, come from individuals selected from populations which have been seed-propagated (most likely selfed), possibly over many generations.

### SSR fingerprint and pedigree information

Of the 224 possible genotypes, 209 (93%) were recovered and 199 (89%) of these cultivars had a unique genotype. An average of 25 SSRs differed between two cultivars (Figure 2), half of the markers used, which demonstrates the high level of SSR discrimination for peach cultivar fingerprinting. Ten groups of cultivars with identical genotype were identified, including 25 cultivars, one group with seven cultivars and nine with two cultivars each. These groups have already been identified [5], but the addition of 34 new markers allowed two cultivars then considered to be possible sports, 'Starcrest' and 'Red Coast', to be separated from groups of genotypes with identical or very similar fingerprints. 'Starcrest' has been described as a sport of 'Springcrest' [15], for which we found differences in six SSRs (BPPCT006, BPPCT015, BPPCT024, BPPCT038, pchgms2 and UDP96-003) with the group of seven cultivars that include most of the know sports of 'Springcrest'. 'Red Coast', was found to be identical to 'Elegant Lady' and 'Rome Star' by [5], but differed in four (BPPCT038, CPPCT026, CPPCT044 and UDP98-025) of the new SSRs studied here. We also identified two further differences between cultivars that were considered as possible SSR mutations [5], one (UDP96-003) between 'Red Top' and the group of 'Lisbeth' and 'June Lady', and the other (CPPCT026) between 'Summer Lady' and the group formed by 'O'Henry' and 'John Henry'. Based on the six putative SSR mutations found in the 28 cultivars having three or less SSR differences between them (the 25 previously described and 'Red Top', 'Summer Lady' and 'Armking'), which we considered putative synonyms or sports, we calculated an overall SSR mutation rate of 2.1 × 10^-3 ^per allele, much lower than our previous estimate of 1.1 × 10^-2 ^[5]. In all cases, only one of the two alleles of the loci differed and each mutation was found at a different locus, a situation compatible with their origin as random mutations.

**Figure 5:**
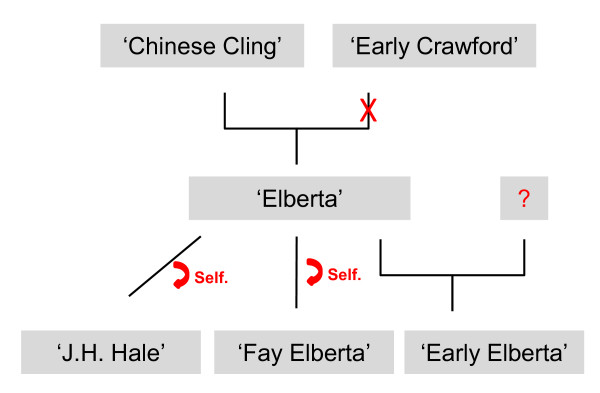
**Pedigree of some of the founder cultivars of the US breeding programs**. Annotations in red indicate our hypothesis based on SSR results.

When considering the accuracy of the pedigree of some of the US founders (Figure 5), our results indicate that 'Elberta' is a seedling of 'Chinese Cling' but not of 'Early Crawford': the genotypes of six of the SSRs of 'Elberta' did not agree with those expected, assuming that it came from this cross. 'Elberta' was obtained from a seed collected from 'Chinese Cling', with 'Early Crawford' trees in the neighbourhood considered the most likely pollen donor [1]. Our data discard this hypothesis, but confirm the involvement of 'Chinese Cling' in the early US breeding programs. 'J.H. Hale', 'Early Elberta' and 'Fay Elberta' are described as open pollinated seedlings from 'Elberta' [15]. This appears to the true for the 'J.H. Hale' genotype, but we found one 'Early Elberta' SSR and two 'Fay Elberta' SSRs that were not in agreement with the original pedigree data. Given the low number of conflicting markers we do not discard that these three cultivars are seedlings of 'Elberta' and that the differences are due to SSR mutations. In this case, SSR genotypes would be compatible with 'J.H. Hale' and 'Fay Elberta' being selfed seedlings from 'Elberta', but that 'Early Elberta' would come from an outcross.

### Population structure, linkage disequilibrium, and prospects for association mapping in peach

We report here the first study of population structure and LD in peach, both crucial aspects for association genetics. Population subdivision can generate spurious associations. The association methods that take population structure into account are insufficient [17,18], with the problem becoming insurmountable when the phenotype of interest is linked to population subdivision. This is the case with the genes that determine peach or nectarine and melting or non-melting flesh, where the search for linked markers cannot be efficiently addressed through association mapping methods. However, each subpopulation shows a high level of variability for other interesting aspects, such as flowering and maturity time, with no correlation between these phenotypes and the subdivision of the varieties into subpopulations (data not shown). The sample size of each subpopulation detected here was small (25 melting peaches, 50 nectarines and 21 non-melting peaches); association methods to localize these characters could be addressed by selecting a set of individuals and applying association statistics that account for population structure in combination with data on the positions of major genes and QTLs for the characters studied obtained through linkage mapping.

To avoid false LD due to population stratification, we calculated the LD in the three presumably unstructured sets of varieties: analysis of all 224 cultivars would result in the 45% of pairs of alleles from loci in different linkage groups being in significant LD (α ≤ 0.05), much higher than the background LD found in each of the three subpopulations (13.9%, 13.4% and 18%, respectively).

In all cases, LD conservation was high and decreased with distance. Raw r^2 ^values and variation of the percentage of loci in LD with distance showed that, in all three populations, LD decayed at 13-15 cM. After this point, between 32-36% of the loci were in significant LD (α ≤ 0.05). When increasing the threshold to α ≤ 0.01, LD decayed at 2-6 cM depending on the population studied. This is similar to that observed in crops such as sugarcane with LD extending to 10 cM [12], cotton with 25 cM LD extension at r^2 ^≥ 0.1 and 5-6 cM at the higher threshold of r^2 ^≥ 0.2 [19], and grapevine where genotypic LD extends to about 16.8 cM [10]. These data support the potential for association mapping of agronomically important traits in peach. The peach genome, with a total genetic distance of approximately 600 cM [20], would require between 100 and 300 evenly spaced polymorphic SSR markers in the subpopulations identified in this paper for a complete genome scan to detect markers associated to major genes or QTLs (considering the high threshold of α ≤ 0.01). Given that the number of SSRs currently mapped in the *Prunus *reference map is 499 [21,22], with the high level of LD conservation there would also be a good chance of finding alleles of major peach genes or QTLs that are associated with alleles of specific SSRs mapping at the same region. This would be useful for marker assisted selection with these alleles and also for the prediction of genotypes of specific gene/QTL regions based on the genotypes of associated markers, of interest for characterising parents in breeding programs and the planning of crossings for specific purposes.

Although SSRs are currently the markers used for genetic analysis in peach, a high number of SNP markers are being developed, providing additional opportunities for high-resolution LD mapping. These studies require knowledge of LD extension and intensity. Tests with simulated data to predict the extent of LD with SNPs based on LD estimates using SSRs [18], indicate that when LD is due to genetic drift, SSR and SNP estimates of LD are similar. However, studies with real data in grape [10,23] and maize [24] reveal that SNPs estimate much lower levels of LD than SSRs. Further studies are required to determine if this is also the case in peach.

The number of individuals analyzed in each subpopulation was small (21 to 50 cultivars) and diversity analysis shows that they were closely related. It has been reported that populations with a broad genetic basis have lower levels of LD than narrow-based populations, so the use of more diverse peach material that exploits the recombination events occurred in its history is likely to result in a population with reduced LD extension for association mapping purposes [25,26]. Chinese peach materials, expected to be more variable [27] with a different recent history under cultivation, may have a lower level of LD conservation. The use of the same or a similar set of markers as those used here would allow comparison of materials of these two origins and estimation of their LD. This could lead to a two-tiered association analysis strategy [28], where populations with high LD could be used for mapping major genes and QTLs or to validate candidate genes and those with a lower LD could be used for fine mapping of specific regions containing genes of interest.

## Conclusions

The study of a large set of SSR markers in a collection of peach cultivars from Europe and North America supported previous data indicating a relatively low level of genetic variation, and a distribution of the molecular variability that places together entries with some of the major commercial fruit characters, such as peaches, nectarines and non-melting flesh peaches. Our results indicate a strong subpopulation structure and a high level of linkage disequilibrium conservation, which may have been caused in part by the fact that most cultivars examined originated from the small set of founders used by the early U.S. breeding programs. These data provide a standard where other molecular variability studies based on sets of cultivars from different origins can be compared, and a foundation for the development of tools for genome-wide surveys of variability based on SNP markers allowing for association genetics studies in this important horticultural crop.

## Methods

### Plant materials and SSR polymorphism detection

Of the set of 224 peach cultivars, most (208) coincide with the 212 previously studied [5]. Eight of the 16 new ones were obtained from the peach germplasm collection of IRTA-Fundació Mas Badia (Girona, Spain), and the 8 remaining ('Admiral Dewey', 'Chinese Cling', 'Early Crawford', 'Elberta', 'Early Elberta', 'Fay Elberta', 'J.H. Hale' and 'Rio Oso Gem') are some of the founder varieties of the USA early breeding programs that were provided by Dr. T. Gradziel of the University of California, Davis. (See Additional file 1 for details on pedigree, origin and fruit morphology of the cultivars studied.)

Genomic DNA was extracted as described in [29] and was analyzed with 50 SSR markers, mapped and evenly distributed in the *Prunus *reference map 'Texas' (almond) × 'Earlygold' (peach) (Additional file 2). Three of the SSRs used were obtained from sweet cherry genomic DNA (PMS2, PS1H3 and PS9f8), two from peach cDNA (EPPCU1090 and pchcms5) one each from sour cherry (PCeGA34) and Japanese plum (CPSCT006) genomic DNA, and the remaining 43 from peach genomic DNA libraries. Amplification and allele detection were carried out as described in [5].

### Variability analysis and mutation rates

The peach variability parameters used were: A, average number of alleles per locus, Ae, effective number of alleles, Ho, observed heterozygosity, He, expected heterozygosity, F, Wright's fixation index, PD, power of discrimination and C, the probability of confusion, i.e. the probability that any two cultivars have identical SSR genotypes by chance alone, considering all loci. These parameters were calculated as in [5]. For diversity analysis, SSR data were scored as 0/0.5/1 (absence/heterozygous allele/homozygous allele). Genetic distances between cultivars were calculated with Nei's parameter [30], implemented by the Simgend procedure of the NTSYSpc V. 2.1 program [31], and a dendrogram was drawn with the same software using the unweighted pair group method average (UPGMA) clustering.

Mutation rates for SSRs were estimated as the ratio between the number of mutated alleles in the group of cultivars that are synonyms or sports and the total number of alleles sampled in this group (100 alleles per cultivar).

### Linkage disequilibrium analysis

Given that the phases between alleles at two heterozygous loci are unknown, we calculated composite linkage disequilibrium (LD) coefficients, a measure reported to have good statistical properties and suggested for routine testing of LD [32]: after removing alleles with frequencies lower than 5% (considering minor allele frequency, MAF ≥ 0.05), composite disequilibrium coefficients (Δ_AB_) between pairs of alleles *A *and *B *at two loci, in either the same or different linkage group, were calculated according to Weir's method [33] with GDA 1.1 software [34]. These calculations were normalized to obtain the inter-allelic Weir's correlation coefficient [33], r^2^_AB, _as in [10]. For this analysis, the null hypothesis of no linkage was tested by comparison with a chi-square statistic with one degree of freedom χ^2 ^= n_*_r^2^_AB_, where n is the number of individuals in the sample (see [35]), and significance thresholds were calculated for α ≤ 0.05 (χ^2^_1df _= 3.841) and α ≤ 0.01 (χ^2^_1df _= 6.635). Therefore, we did not correct the LD estimate for sample size since it was already accounted for by the significance value. For the curve that represents the observed r^2 ^values, we used the average values for each of 4 subsets of equal number of pair comparisons covering adjacent intervals along distance.

### Population Structure analysis

Population structure was studied with the *Structure *v.2 [36] software. This program uses a clustering method that identifies *K *subgroups of individuals with distinctive allele frequencies. Individuals can be members of multiple subpopulations with a different coefficient, with the sum of all being equal to 1. To check for population stratification in our sample we ran the program under the admixture model assumption with correlated alleles. The run used 10^6 ^interactions after a burn-in of 10^5 ^for a value of *K *ranging from 2 to 8.

## Authors' contributions

MJA and PA conceived and designed this study and wrote the manuscript. MJA obtained molecular marker data and performed the statistical analysis. EKA obtained molecular marker data and participated in the variability analysis. WH contributed in the molecular data uptake and data analysis. All authors read and approved the final manuscript.

## Supplementary Material

Additional file 1 **Description of the 224 peach cultivars used**. (a) Most pedigree data were obtained from Okie's Handbook [15]; (b) - Unknown; (c) First letter: P = peach, N = nectarine, F = flat peach. Second letter: W = white, Y = yellow. Third letter: N = non-melting flesh, M = melting flesh; (d) Observed heterozygosity (Ho) Click here for file

Additional file 2 **Map position of the SSRs used in this study in the *Prunus *reference map 'Texas' × 'Earlygold'**. Click here for file
