# Supplementary material for: Genetic variation, population structure and linkage disequilibrium in peach commercial varieties
Source: BMC Genet. 2010 Jul 20;11:69. doi: 10.1186/1471-2156-11-69 (PMC2915947; doi:10.1186/1471-2156-11-69)
Supplement: Additional file 1 — Description of the 224 peach cultivars used. (a) Most pedigree data were obtained from Okie's Handbook [15]; (b) - Unknown; (c) First letter: P = peach, N = nectarine, F = flat peach. Second letter: W = white, Y = yellow. Third letter: N = non-melting flesh, M = melting flesh; (d) Observed heterozygosity (Ho) [file 1471-2156-11-69-S1.PDF]

Cultivar	Pedigree[(a)]	Breeder (Country)	Fruit traits[(b)]	Ho[(c)]
86-6	-	Zaiger - USA	NYN	0.44
Admiral Dewey	Alexander op.	-	PYM	0.11
Alexandra	Fayette x Royal Gold	Zaiger's Genetics (Zaiger) - USA	PWM	0.60
Aline	O'Henry x Giant Babcock	Zaiger - USA	PWM	0.53
Andross	Dix 5A-1 x Fortuna	UCD - USA	PYN	0.17
Armking	(Palomar x Springtime) x (Palomar x Springtime)	Armstrong Nurseries (Armstrong) - USA	NYM	0.37
Armking-2	-	Armstrong - USA	NYM	0.50
Armking-3	-	Armstrong - USA	NYM	0.38
ASF_R7a68Nj	-	-	NYM	0.20
Auberge	-	Traditional cultivar (TC) - Spain	PWN	0.04
Auberge Blanc	-	TC - Spain	PWN	0.00
Auberge Ferran	-	TC - Spain	PWN	0.13
Auberge Marino	-	TC - Spain	PWN	0.22
August Orebrad	(Red Diamond x Autumn Free) F_2	Bradford Frams (Bradford) - USA	NYN	0.28
August Queen	Stark Red Gold x Snow Queen	IPSA, Faenza (IPSA) - Italy	NWM	0.27
Aurora Grand	Aurelio Grand op.	Bradford - USA	NYM	0.14
Autumn Free	Sun Grand x Gold King	Bradford - USA	NYM	0.28
Baby Gold-8	PI35201 x Ambergem	Rutgers University and New Jersey A.E.S., New Brunswick (RU-NJ) - USA	PYN	0.52
Babygold-7	(Lemon Free x PI35201) x NJ196	RU-NJ - USA	PYN	0.61
Betty	Redwing x W6-120	Zaiger - USA	PWM	0.54
Big Top	-	Zaiger - USA	NYM	0.33
Bigsun	-	Maillard - France	PYM	0.14
Binaced	-	Servicio de Investigación Agraria, Zaragoza - Spain	PWN	0.20
Bolero	Cresthaven x Flamecrest	University of Bologna, ICA - CMVF, Bologna (ICA-CMVF) - Italy	PYM	0.37
Bonbon	Honey Gold op.	Maillard - France	NYM	0.34
Calabacero	-	TC - Spain	PYN	0.39
Calante	-	TC - Spain	PYN	0.14
Canongi	-	TC - Spain	PYN	0.39
Carolina	Fla.3-4N x Sunred	University of Florida, Gainesville (UFG) - USA	NYM	0.51
Carson	Maxine x Leader	U.S.D.A. Palo Alto, California - USA	PYN	0.32
Casaboi	-	Univeristy of Florida/Selección Plantas Sevilla (UFG -SPS) - USA/Spain	PWM	0.43
Casarob	-	UFG -SPS - USA/Spain	PWM	0.57
Catherina	NJC95 x D42-13w	RU-NJ - USA	PYN	0.55
Chinese Cling	-	Imported from China	PWN	0.56
Christalrose	NB570 x Favols	Escande - France	NYM	0.36
Cofrentes-3	-	TC - Spain	PYN	0.04
Cofrentes-5	-	TC - Spain	PYN	0.25
Cofrentes-6	-	TC - Spain	PYN	0.00
Corine	-	Escande - France	PYM	0.49
Cotigua	-	TC - Spain	PYN	0.14
Cresthaven	Kalhaven x SH309	Michigan State Univeristy, East Lansing (MSU) - USA	PYM	0.22
Crimson Lady	(Red Diamond x Springcrest) x Seedling	Bradford - USA	PYN	0.51
Daisy	Rhone Gold x Redwing	Zaiger - USA	PWM	0.58
Delice	-	Maillard - France	PYM	0.18
Dellys	-	Escande - France	NWM	0.27
Dolores	O'Henry x Early Babcock	Zaiger - USA	PWM	0.15
Douceur	-	Maillard - France	PWM	0.44
Duchessa d'Este	(Mayflower x Amsden) op.	Scanavini - Italy	PWM	0.56
Early Crawford	-	Crawford (1841)	PYM	0.29
Early Elberta	Elberta op.	-	PWM	0.67
Early Giant	-	Zaiger - USA	NYM	0.13
Early Maycrest	Maycrest mutation	Toeus, Ridley, California - USA	PYM	0.57
Early O'Henry	O'Henry op.	Grant Merrill (Merrill) -USA	PYM	0.29
Early Orebrad	-	Bradford - USA	NYM	0.46
Early Sun Grand	Sun Grand op.	Bradford - USA	NYM	0.09
Elberta	Chinese Cling op (perhaps x Early Crawford)	S. H. Rumph - USA	PYM	0.80
Elegant Lady	Early O'Henry x July Lady	Merrill - USA	PYM	0.40
Escarolita	-	TC - Spain	PYN	0.12
Escarolita Ferran	-	TC - Spain	PYN	0.14
Etoile	-	Maillard - France	PYM	0.06
Evaisa	-	TC - Spain	PYN	0.04
Fairlane	P60-38 x Fantasia	U.S.D.A. Fresno, California (Fresno) - USA	NYM	0.29
Fantasia	Gold King x P101-24	Fresno - USA	NYM	0.35
Fantasie	-	Maillard - France	PYM	0.17
Fay Elberta	Elberta op.	-	PYM	0.49
Federica	NJC11 x (NJ13232 X Cherryred)	RU-NJ/ISF - USA/Italy	PYN	0.17
Festina	-	Escande - France	NWM	0.09
Fidelia	(O'Henry x Giant Babcock) x (May Grand x Sam Houston)	Zaiger - USA	PWM	0.46
Fidelia Ruth	Fidelia mutation	IRTA-Fundació Mas Badia, Girona (IRTA) TC - Spain	NWM	0.43
Fire Gold	-	Zaiger - USA	NYM	0.41
Fire Red	B8-12 self.	UCD - USA	PYM	0.21
Fireking	-	Armstrong - USA	NYM	0.43
Flaminia	Fayette x Fairtime	ISF - Italy	PYM	0.30
Flavor Crest	P53-68 x FV89-14	Fresno - USA	PYM	0.46
Flavor Gold	Rhone Gold x Royal Gold	Zaiger - USA	NYM	0.48

Cultivar	Pedigree[a]	Breeder (Country)	Fruit traits[b]	Ho[c]
Flavortop	Fairtime op.	Fresno - USA	NYM	0.30
Flavour Giant	-	Zaiger - USA	NWM	0.21
Flordaking	Fla.9-67 x Early Amber	UFG - USA	PYM	0.43
Flordastar	Flordagold x EarliGrande	UFG - USA	PYM	0.44
Glady's	-	Zaiger - USA	PWM	0.47
Glenna	-	Zaiger - USA	PWM	0.45
Gorga	-	TC - Spain	PYN	0.22
Gratia	-	Zaiger - USA	PWM	0.33
Hardired	Lexington x NJN32	Ontario, Agriculture Canada, Harrow - Canada	NYM	0.47
Hermione	-	Zaiger - USA	PWM	0.49
Independence	Red King op.	Fresno - USA	NYM	0.00
Isabella D'Este	-	Lodi, Ferrara - Italy	PWM	0.48
J.H.Hale	Chance Sdlg, possible of Elberta	J. H. Hale	NYM	0.43
Jeronimo	-	TC - Spain	PYN	0.30
Jesca	Sel. from Amarillos Tardios de Calanda	TC - Spain	PYN	0.31
John Henry	O'Henry mutation	California (USA)	PYM	0.41
July Lady	J.H.Hale X x Merril Gem F_2	Merrill - USA	PYM	0.23
June Crest	Fayette x Mexican Seedling	Zaiger - USA	PYM	0.41
June Lady	Fortynine x Gemfree	Merrill - USA	PYM	0.41
Kay Grand	Red Grand x Stark Sunglo	Bradford - USA	NYM	0.18
Lamone	Babygold 6 x Shasta	ICA-CMVF - Italy	PYN	0.43
Large White	-	U.S.D.A. Baton Rouge, Lousiana - USA	PWM	0.54
Lisbeth	Fortyniner x Gemfree	Merrill - USA	PYM	0.35
Lucie	-	Bradford - USA	PYM	0.40
Magique	-	-	NWM	0.21
Maria Aurelia	Stark Redgold self.	University of Florence, DOFI, Florence (DOFI) - Italy	NYM	0.30
Maria Bianca	Honey Dew Hale x Michelini	DOFI - Italy	PWM	0.27
Maria Delizia	Cesarini op.	DOFI - Italy	PWM	0.19
Maria Emilia	May Grand self.	DOFI - Italy	NYM	0.10
Maria Laura	Flavortop self.	DOFI - Italy	NYM	0.15
Maruja	-	TC - Spain	PYN	0.31
Maybelle	Armking mutation	Parnagian, Fowler, California - USA	NYM	0.38
Maycrest	Springcrest mutation	Minami, Reedley, California - USA	PYM	0.59
MB-3	-	IRTA - Spain	PWN	0.06
Michelini	-	Michelini, Savona - Italy	PWM	0.55
Mid Silver	-	Zaiger - USA	NWM	0.18
Moon Grand	(Red Grand x Early Sun Grand) F_2	Bradford - USA	NYM	0.10
Morsiani 51	Sirio x Superstar	P.L.Morsiana, Ravenna - Italy	NYM	0.32
N-1534/70	-	Zaiger - USA	NYM	0.49
Nectaprima	Zaitabo x Armking	Maillard - France	NYM	0.30
Nectariane	Andano x Zaitabo	Maillard - France	NYM	0.36
Nectaross	Stark Redgold x Le Grand	ISF, Rome (ISF) - Italy	NYM	0.30
Niagara	Red Grand x Early Sun Grand	Bradford - USA	NYM	0.37
NJC-97	-	RU-NJ - USA	PYN	0.27
NJN-76	-	RU-NJ - USA	NYN	0.17
O'Henry	Merrill Bonanza op.	Merrill - USA	PYM	0.41
P-1319	-	Zaiger - USA	PWM	0.45
P-86-124	-	Zaiger - USA	PWM	0.44
P-88-206	-	UFG-SPS - USA/Spain	PWM	0.59
Pacific Star	May Grand op.	Weinberger, Fresno, California - USA	NYM	0.18
Paraguayo Delfin	-	TC - Spain	FWM	0.60
Peret	-	TC - Spain	PYN	0.26
Peret Ferran	-	TC - Spain	PYN	0.22
Peret Marino	-	TC - Spain	PYN	0.22
Perfect Delight	Firebrite x Stark Redgold	Zanzi, Ferrara - Italy	NYM	0.23
Pretty Rubis	-	-	NWM	0.41
Queen Crest	Maycrest mutation	Balakian Reedley, California - USA	PYM	0.57
Queen Giant	-	Zaiger - USA	NWM	0.38
Queen Lady	July Lady x 59-125	Merrill - USA	PYM	0.58
Queen Ruby	-	Zaiger - USA	NWM	0.32
Red Coast	-	C.L.C. Ferrara - Italy	PYM	0.32
Red Delight	6W120 op.	Zaiger - USA	NYM	0.16
Red Diamond	Red Grand x Early Sun Grand	Bradford - USA	NYM	0.20
Red Fair	-	Zaiger - USA	NYM	0.27
Red Moon	Flamecrest op.	C.I.V. Ferrara - Italy	PYM	0.35
Red Pearl	-	Zaiger - USA	NYM	0.23
Red Silver	-	Zaiger - USA	NWM	0.42
Red Top	Sunhigh x July Elberta	Fresno - USA	PYM	0.34
Red Valley	Elegant Lady op.	C.I.V. Ferrara - Italy	PYM	0.22
Redhaven	Halehaven x Kalhaven	MSU - USA	PYM	0.47
Redwing	Babcock x Stensgaard July Elberta	Armstrong - USA	PWM	0.55
Rich Lady	Amparo op.	Zaiger - USA	PYM	0.36
Rich May	[(May Grand x Peach) x Sam Houston] x (Tasty Gold x Maycrest)	Zaiger - USA	PYM	0.29
Rio Oso Gem	Late Crawford op.	-	PYM	0.31
Rome Star	(Fayette x Stark Redgold) op.	ISF - Italy	PYM	0.40

Cultivar	Pedigree[(a)]	Breeder (Country)	Fruit traits[(b)]	Ho[(c)]
Romea	Catherina op.	ISF - Italy	PYN	0.49
Rose Diamond	Early Diamond x Dwarf Peach	Bradford - USA	NYM	0.23
Rosered	-	-	NWM	0.17
Rosired-3	Southland self.	ICA-CMVF - Italy	PYM	0.10
Royal Gem	-	Zaiger - USA	PYM	0.21
Royal Giant	Red Grand x Seedling	Zaiger - USA	NYM	0.27
Royal Glory	May Grand op.	Zaiger - USA	PYM	0.60
Royal Moon	-	Zaiger - USA	PYM	0.54
Royal Prince	-	Zaiger - USA	PYM	0.45
Ruby Gem	-	Zaiger - USA	NWM	0.37
Ruby Rich	-	Zaiger - USA	PYM	0.17
San Lorenzo	-	TC - Spain	PYN	0.35
Sarah	-	Maillard - France	PWM	0.49
Seduction	-	Maillard - France	PYM	0.21
Sensation	-	Maillard - France	PYM	0.16
September Orebrad	(Red Free x Tom Grand) x Sparkling Red	Bradford - USA	NYM	0.21
September Queen	Stark Red Gold x Snow Queen	IPSA - ITaly	NWM	0.23
Sibelle	-	Maillard - France	PYM	0.12
Silver Belle	-	Zaiger - USA	NWM	0.43
Silver Gem	May Grand x Chance Seedling	Zaiger - USA	NWM	0.40
Silver King	Armking mutation	Prim - France	NWM	0.36
Silver Late	-	Zaiger - USA	NWM	0.47
Silver Ray	(Stark Redgold x Snow Queen) op.	V.Ossani, FaViFrut, Faenza (FaViFrut) - Italy	NWM	0.39
Silver Rome	Stark Redgold x Snow Queen	FaViFrut - Italy	NWM	0.33
Silver Star	August Queen op.	FaViFrut - Italy	NWM	0.22
Silvery	-	Zaiger - USA	NWM	0.44
Sirio	Flamekist x Fantasia	ISF - Italy	NYM	0.42
Snow Queen	-	Armstrong - USA	NWM	0.47
Snowflame	(Nectar op.) op.	James F. Doyle - USA	PYM	0.20
Snowred	-	Escande - France	NWM	0.19
Spring Bright	May Diamond x Seedling	Bradford - USA	NYM	0.33
Spring Lady	-	Merrill - USA	PYM	0.55
Spring Red	Summer Grand op.	Bradford - USA	NYM	0.16
Springbelle	-	Batistini - Italy	PYM	0.55
Springold	FV89-14 x Springtime	U.S.D.A. Byron and Fort Valley, Georgia (Byron) - USA	PYM	0.57
Starcrest	Springcrest mutation	Chapus and Veauvy - France	PYM	0.47
Stark Redgold	Sun Grand op.	Bradford - USA	NYM	0.35
Starlite	FV89-14 x Springtime	Byron - USA	PWM	0.45
Summer Grand	Late Le Grand x Early Sun Grand	Bradford - USA	NYM	0.46
Summer Lady	O'Henry mutation	California - USA	PYM	0.42
Summer Rich	-	Zaiger - USA	PYM	0.42
Suncrest	Alamar x Gold Dust	Fresno - USA	PYM	0.43
Super Crimson Gold	Zee Gold x Early Sun Grand	Zaiger - USA	NYM	0.44
Superqueen	Stark Redgold x Snow Queen	IPSA - ITaly	NWM	0.28
Superstar	Summer Grand op.	Sun World International - USA	NYM	0.23
Sweet Cap	-	Maillard - France	FWM	0.46
Sweet Lady	Stark Redgold op.	CO.VI.CO, Faenza - Italy	NYM	0.37
Sweet Red	Stark Redgold op.	CO.VI.CO, Faenza - Italy	NYM	0.33
Symphonie	Early O'Henry op.	Maillard - France	PYM	0.35
Tardibelle	-	Maillard - France	PYM	0.13
Tasty Free	Red Free x Autumn Gold	Bradford - USA	NYM	0.34
Tendresse	-	Maillard - France	PWM	0.35
Tirrenia	Vivian x Federica	ISF - Italy	PYN	0.38
Top Lady	-	Merrill - USA	PYM	0.18
Topcrest	Fayette x Mexican Sdlg	Zaiger - USA	PYM	0.27
Venus	Stark Redgold x Flamekist	ISF - Italy	NYM	0.37
Very Good	-	-	PYM	0.17
Villa Ada	Catherina op.	ISF - Italy	PYN	0.35
Villa Doria	Catherina op.	ISF - Italy	PYN	0.29
Villa Giulia	Catherina op.	ISF - Italy	PYN	0.30
Vista Rich	Rich Lady op.	Zaiger - USA	PYM	0.33
Voluptia	-	ISF - Italy	PWM	0.44
Weinberger	F100-62 x Red June	ISF - Italy	NYM	0.20
Weinberger 5199	-	Italy	PYM	0.38
White Lady	(O'Henry x Giant Babcock) x (May Grand x Sam Houston)	Zaiger - USA	PWM	0.37
Zee Glo	Red Grand op. x (Sun Grand x Merrill Gem)	Zaiger - USA	NYN	0.41
Zee Lady	O'Henry x June Lady	Zaiger - USA	PYM	0.38
Zincal 5	(Fayette x Maygrand) F2	Zaiger - USA	NYM	0.20
Zinege	-	Zaiger - USA	NWM	0.45
Zinepre	-	Zaiger - USA	NYM	0.17
Zisearl	-	Zaiger - USA	NYM	0.45
Zisecan	-	Zaiger - USA	PYM	0.41
Zisesil	-	Zaiger - USA	NWM	0.42
Zisesun	-	Zaiger - USA	NYM	0.36
