# Supplementary material for: Genetic variation, population structure and linkage disequilibrium in peach commercial varieties
Source: BMC Genet. 2010 Jul 20;11:69. doi: 10.1186/1471-2156-11-69 (PMC2915947; doi:10.1186/1471-2156-11-69)
Supplement: Additional file 2 — Map position of the SSRs used in this study in the Prunus reference map 'Texas' × 'Earlygold'. [file 1471-2156-11-69-S2.PDF]

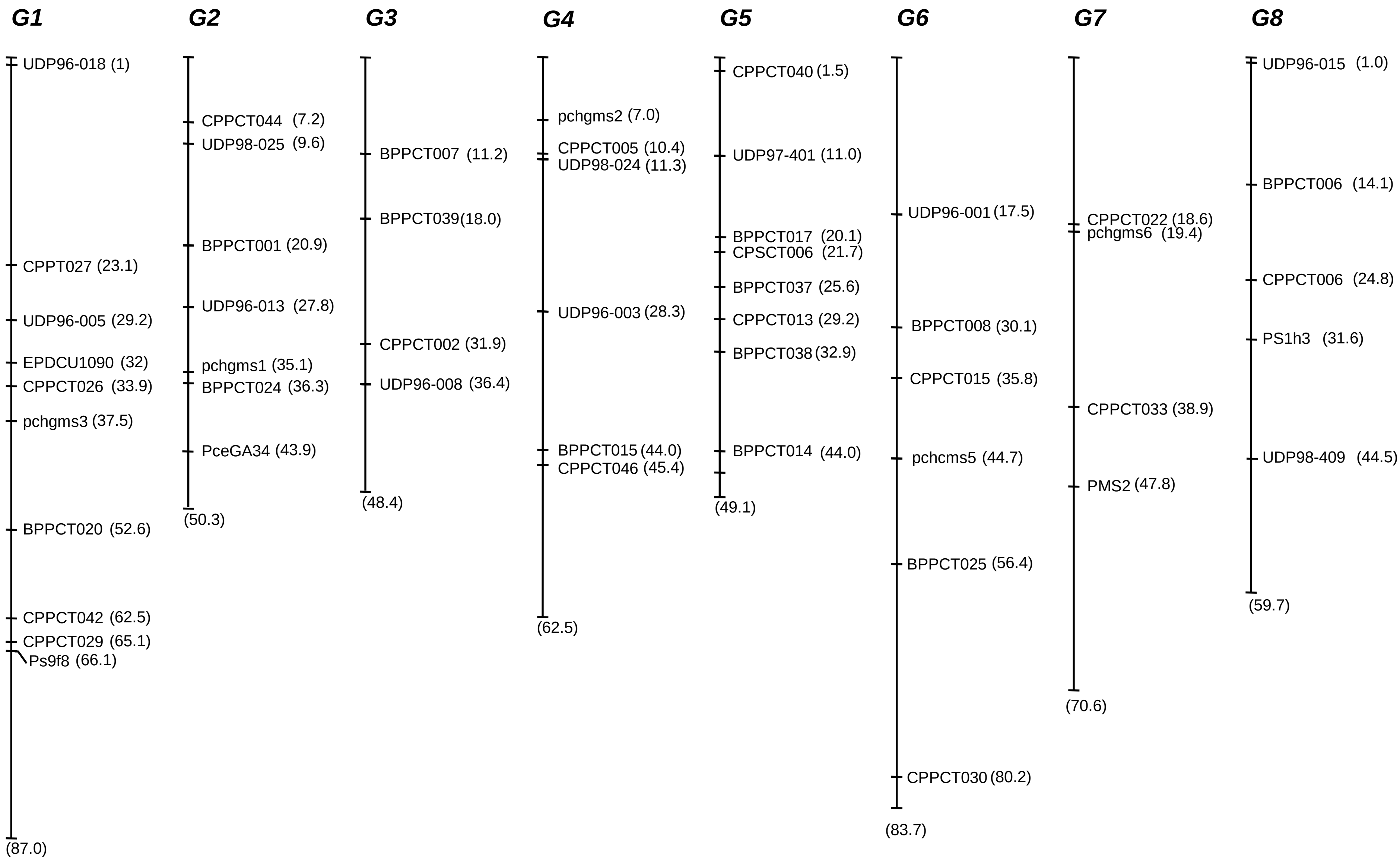

G1
UDP96-018 (1)
CPPT027 (23.1)
UDP96-005 (29.2)
EPDCU1090 (32)
CPPCT026 (33.9)
pchgms3 (37.5)
BPPCT020 (52.6)
CPPCT042 (62.5)
CPPCT029 (65.1)
Ps9f8 (66.1)
(87.0)
G2
CPPCT044 (7.2)
UDP98-025 (9.6)
BPPCT001 (20.9)
UDP96-013 (27.8)
pchgms1 (35.1)
BPPCT024 (36.3)
PceGA34 (43.9)
(50.3)
G3
BPPCT007 (11.2)
BPPCT039(18.0)
CPPCT002 (31.9)
UDP96-008 (36.4)
(48.4)
G4
pchgms2 (7.0)
CPPCT005 (10.4)
UDP98-024 (11.3)
UDP96-003 (28.3)
BPPCT015 (44.0)
CPPCT046 (45.4)
(62.5)
G5
CPPCT040 (1.5)
UDP97-401 (11.0)
BPPCT017 (20.1)
CPSCT006 (21.7)
BPPCT037 (25.6)
CPPCT013 (29.2)
BPPCT038 (32.9)
BPPCT014 (44.0)
(49.1)
G6
UDP96-001 (17.5)
BPPCT008 (30.1)
CPPCT015 (35.8)
pchcms5 (44.7)
BPPCT025 (56.4)
CPPCT030 (80.2)
(83.7)
G7
CPPCT022 (18.6)
pchgms6 (19.4)
CPPCT033 (38.9)
PMS2 (47.8)
(70.6)
G8
UDP96-015 (1.0)
BPPCT006 (14.1)
CPPCT006 (24.8)
PS1h3 (31.6)
UDP98-409 (44.5)
(59.7)
